# Listening effort and accented speech

**DOI:** 10.3389/fnhum.2014.00577

**Published:** 2014-08-05

**Authors:** Kristin J. Van Engen, Jonathan E. Peelle

**Affiliations:** ^1^Department of Psychology, Washington University in St. LouisSt. Louis, MO, USA; ^2^Department of Otolaryngology, Washington University in St. LouisSt. Louis, MO, USA

**Keywords:** listening effort, speech comprehension, accent, speech perception, speech perception in noise

Understanding spoken language requires mapping acoustic input onto stored phonological and lexical representations. Speech tokens, however, are notoriously variable: they fluctuate within speakers, across speakers, and in different acoustic environments. As listeners, we must therefore perceive speech in a manner flexible enough to accommodate acoustic signals that imperfectly match our expectations. When these mismatches are small, comprehension can proceed with minimal effort; when acoustic variations are more substantial, additional cognitive resources are required to process the signal. A schematic model of speech comprehension is shown in Figure 1, emphasizing that different degrees of acoustic mismatch will require varying levels of cognitive recruitment. Recent research increasingly supports a critical role for executive processes—such as verbal working memory and cognitive control—in understanding degraded speech (Wingfield et al., 2005; Eckert et al., 2008; Rönnberg et al., 2013). However, to date, the literature has focused on sources of increased acoustic challenge that originate in the listener (hearing loss) or in the listening environment (background noise). Largely unexplored are the cognitive effects of accented speech (i.e., speech produced by a speaker who does not share a native language or dialect with the listener), a ubiquitous source of variability in speech intelligibility. Here we argue that accented speech must also be considered within a framework of listening effort.

**Figure 1:**
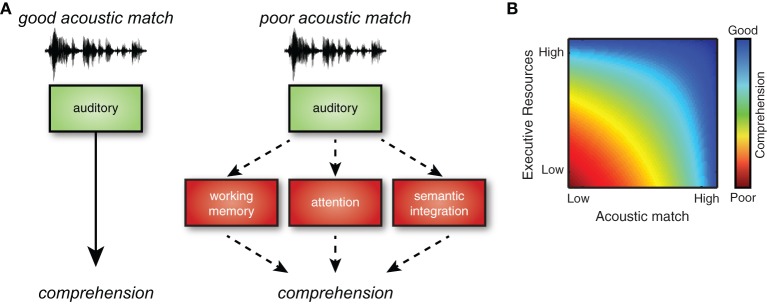
**(A)** Speech signals that match listeners' perceptual expectations are processed relatively automatically, but when acoustic match is reduced (for example, due to noise or unfamiliar accents), additional executive resources are needed to compensate. **(B)** Executive resources are recruited in proportion to the degree of acoustic mismatch between incoming speech and listeners' representations. When acoustic match is high, good comprehension is possible without executive support. However, as the acoustic match becomes poorer, successful comprehension cannot be accomplished unless executive resources are engaged. Not shown is the extreme situation in which acoustic mismatch is so poor that comprehension is impossible.

## Listening effort

Recent years have seen an increasing focus on the cognitive effects of acoustic challenge during speech comprehension (Mattys et al., 2012). A common theme is that when speech is acoustically degraded, it deviates from what listeners are used to (i.e., stored phonological and lexical representations), resulting in a mismatch between expectation and percept (Sohoglu et al., 2012, 2014). As a result, listeners must recruit additional cognitive resources to make sense of degraded speech (Rönnberg et al., 2013). Given that listeners' cognitive resources are limited, at some point the allocation of cognitive resources to resolve acoustic challenge will begin to impinge upon other types of behavior. Indeed, even mild hearing loss has been shown to impact syntactic processing (Wingfield et al., 2006), running memory for speech (McCoy et al., 2005), and subsequent memory for short stories (Piquado et al., 2012). Further support for the connection between acoustic and cognitive processing comes from the fact that behavioral challenges are exacerbated in older adults due to age-related cognitive decline (Wingfield et al., 2005).

If increased executive processing is required to deal with acoustic challenge, the effects should not only be apparent in listeners with hearing loss, but in listeners with good hearing in cases of external auditory interference. Consistent with this view, acoustic distortion reduces the episodic recall of word pairs (Heinrich and Schneider, 2011) or word lists (Rabbitt, 1968; Cousins et al., 2014). Conversely, increasing speech clarity through the use of listener-oriented speech facilitates recognition memory for spoken sentences (Van Engen et al., 2012). Thus, listening effort appears to be a general consequence of challenging speech signals, in which acoustic mismatch can arise due to either internal factors such as hearing loss, or external factors such as background noise.

Functional neuroimaging studies have begun to link these additional executive resources to specific neural systems by identifying increased neural activity resulting from acoustic challenge during speech comprehension (Davis and Johnsrude, 2003; Eckert et al., 2008, 2009; Adank, 2012; Hervais-Adelman et al., 2012; Obleser et al., 2012; Erb et al., 2013). These increases in neural activity frequently involve areas not seen during “normal” speech comprehension—such as frontal operculum, anterior cingulate, and premotor cortex—consistent with listeners' recruitment of additional executive resources to cope with acoustic challenge. Evidence that these increases in brain activity are task-relevant comes from the fact that they vary as a function of attention (Wild et al., 2012), and modulate behavioral performance on subsequent trials (Vaden et al., 2013).

Taken together, then, there is clear evidence that when speech is acoustically degraded, listeners must rely on additional cognitive resources, supported by an extensive network of brain regions. This general principle has been shown in listeners with hearing loss and in good-hearing listeners presented with acoustically degraded materials. In the next section we consider how these findings may play out in the context of understanding accented speech.

## Listening effort and accented speech

If acoustic deviation from stored phonological/lexical representations is indeed the primary cause of increased listening effort, then speech produced in an unfamiliar accent (whether a regional accent or a foreign accent) should similarly affect not only speech intelligibility, but also the efficiency and accuracy of linguistic processing, and memory for what has been heard. Furthermore, accented speech would also be expected to involve the recruitment of compensatory executive resources.

Foreign-accented speech, for example, is characterized by systematic segmental and/or suprasegmental deviations from native language norms. Naturally, these mismatches can lead to a reduction in the intelligibility of the speech (Gass and Varonis, 1984; Munro and Derwing, 1995; Bent and Bradlow, 2003; Burda et al., 2003; Ferguson et al., 2010; Gordon-Salant et al., 2010a,b). However, even when foreign-accented speech is fully intelligible to listeners (i.e., they can correctly repeat or transcribe it), processing it requires more effort than processing native accents: listeners report that accented speech is more difficult to understand (Munro and Derwing, 1995; Schmid and Yeni-Komshian, 1999), and it is processed more slowly (Munro and Derwing, 1995; Floccia et al., 2009) and comprehended less well than native-accented speech (Anderson-Hsieh and Koehler, 1988; Major et al., 2002). Similar effects have been observed for unfamiliar regional accents: Adank et al. (2009) have shown, for example, that listeners' response times and error rates on a semantic verification task (i.e., responding to simple true/false questions spoken with different accents) are higher for speech produced in an unfamiliar regional accent. (For a review of the costs associated with processing accented speech across the lifespan, see Cristia et al., 2012.)

The behavioral consequences of listening to accented speech, therefore, include reductions in intelligibility, comprehensibility, and processing speed—all effects that mirror those seen under conditions involving acoustic degradation. To date, there are few functional neuroimaging studies investigating whether increased brain activity is also seen in response to accented speech, although published accounts suggest this is indeed the case (Adank et al., 2012). In general, we would expect that when listening to accented speech, people will recruit comparable executive resources as when listening to other forms of degradation. This would be consistent with increased activity in regions of premotor cortex, inferior frontal gyrus, and the cingulo-opercular network.

That being said, it is important to acknowledge that mismatches between incoming signals and stored representations can arise by different mechanisms. For degraded speech—including steady-state background noise, hearing impairment, or aided listening—listeners experience a loss of acoustic information. This loss is systematic insofar as it involves the inaudibility of a particular portion of the acoustic signal. In accented speech, there are systematic mismatches between the incoming signal and listeners' expectations, but these arise through phonetic and phonological deviations rather than through signal loss. The degree to which the source of acoustic mismatch affects the type and degree of compensatory cognitive processing required for understanding speech remains an open question. It could be that degraded and accented speech require similar types of executive compensation, and thus both neural and behavioral consequences are largely similar. A second option is that although listeners show similar behavioral consequences to these two types of speech, they are obtained through the use of different underlying neural mechanisms. Finally, there may be differences in both the neural and behavioral consequences of degraded compared to accented speech, or between different types of accented speech. The available preliminary evidence suggests a possible dissociation at the neural level, with different patterns of recruitment for speech in noise compared to accented speech (Adank et al., 2012), and in regional compared to foreign accents (Goslin et al., 2012). However, additional data are needed, and the results may also depend on the level of spoken language processing being tested (Peelle, 2012), task demands, and other factors that determine cognitive challenge for listeners.

## Additional contributions to listening effort

There are undoubtedly a number of additional influences on the perception of accented speech which may not be relevant for acoustically degraded speech. These include familiarity with an accent (Gass and Varonis, 2006), cultural expectations (Hay and Drager, 2010), and intrinsic listener motivation (Evans and Iverson, 2007). Acoustic familiarity may be specifically related to speech, or simply reflect the experience of a particular listener (Holt, 2006). Together, this confluence of factors can interact with acoustic mismatch to determine the degree of perceptual effort experienced by listeners.

## Why do the cognitive consequences of accented speech matter?

If understanding accented speech indeed requires additional cognitive support, then listeners are likely to have greater difficulty not only understanding their accented interlocutors (i.e., reduced intelligibility), but also comprehending and remembering what they have said, and possibly in managing other information or tasks while listening to accented speech. Given the ubiquity of accented speakers (both foreign and regional) in contemporary society, the practical implications of these problems are wide-ranging. Consider, for example, classrooms with foreign-accented teachers or medical settings where patients and medical personnel who *do* share a language may nevertheless *not* speak with similar accents. In such situations the compensatory cognitive processing that can often (though not always) maintain high intelligibility between speakers and listeners may still come at a cost to listeners' ability to encode critical information. Within the context of a broader framework for effortful listening, it is clear that such challenges will be further exacerbated in the frequently-encountered case of acoustic degradation (such as from background noise or hearing loss), where mismatches between incoming speech and listeners expectations can arise from *both* loss of acoustic information and from distortion due to accent. It has been observed, for example, that noisy or reverberant listening environments disproportionately reduce the intelligibility of foreign-accented speech as compared to native-accented speech (Van Wijngaarden et al., 2002; Rogers et al., 2006).

An important point is that effortful listening is not an all-or-none phenomenon; rather, the level of cognitive compensation required will depend on the degree of acoustic mismatch in any given listening situation. A relatively mild accent, for example, or one that is highly familiar to a particular listener, can be well understood and require little to no additional effort. Furthermore, we know that listeners can rapidly adapt to both foreign-accented speech (Clarke and Garrett, 2004; Bradlow and Bent, 2008; Sidaras et al., 2009; Baese-Berk et al., 2013) and speech produced in unfamiliar regional accents (Clopper and Bradlow, 2008; Maye et al., 2008; Adank and Janse, 2010). Assuming that understanding accented speech is cognitively challenging due to mismatches between signals and listener expectations, as suggested by the general model of effortful listening presented here, it follows that such perceptual adaptation to an accent will decrease listening effort, and thereby *increase* functional cognitive capacity: Adaptation effectively reduces the mismatch between incoming speech and listener expectations, thus lowering the demand for compensatory executive processes (Figure 1). Auditory training with accented speech may therefore not only be useful for improving intelligibility, but also for increasing listeners' cognitive capacity^1^.

## Conclusions

When speech does not conform to listeners' expectations, additional cognitive processes are required to facilitate comprehension. In the case of acoustic degradation, it is increasingly accepted that this type of effortful listening can interfere with subsequent attention, language, and memory processes. Here we have argued that accented speech shares critical characteristics with acoustically degraded speech, and that considering the cognitive consequences of acoustic mismatch is critical in understanding how listeners deal with accented speech.

### Conflict of interest statement

The authors declare that the research was conducted in the absence of any commercial or financial relationships that could be construed as a potential conflict of interest.

## Footnotes

^1^It is possible that improvements over the course of perceptual learning also rely to some degree on executive processes. So although the most straightforward prediction is that perceptual adaption will reduce the demand on executive resources, the degree to which this actually happens is an empirical question.
